# A Practical Introduction to Utilising Uncertainty Information in the Analysis of Essential Climate Variables

**DOI:** 10.1007/s10712-025-09906-7

**Published:** 2025-09-13

**Authors:** Adam C. Povey, Claire E. Bulgin, Alexander Gruber

**Affiliations:** 1 School of Physics and Astronomy, University of Leicester, University Road, Leicester, LE1 7RH UK; 2 National Centre for Earth Observation, University of Leicester, 92 Corporation Road, Leicester, LE4 5SP UK; 3 Department of Meteorology, University of Reading, Whiteknights Campus, Reading, RG6 6ET UK; 4 National Centre for Earth Observation, University of Reading, Whiteknights Campus, Reading, RG6 6ET UK; 5 Department of Geodesy and Geoinformation, Technische Universität Wien (TU Wien), Wiedner Hauptstrasse 8–10, 1040 Vienna, Austria

**Keywords:** Uncertainty, Essential climate variables, Dataset aggregation, Representativeness, Validation, Data assimilation

## Abstract

An estimate of uncertainty is essential to understanding what information is conveyed by data and how it relates to the wider context of what one intended to measure. It can be difficult to know how to use uncertainty during the analysis of environmental data and the best way to present that information within a dataset. In many common uses, such as calculating statistical significance, it is easy to make mistakes due to incomplete or inappropriate use of the available uncertainty information. Uncertainty is itself uncertain, such that many practical or empirical solutions are available when a comprehensive uncertainty budget is impractical to produce. This manuscript collects actionable guidance on how uncertainty can be used, presented, and calculated when working with essential climate variables (ECVs). This includes qualitative discussions of the utility of uncertainties, explanations of common misconceptions, advice on presentation style, and plain descriptions of the essential equations. Selected worked examples are included on the propagation of uncertainties, particularly for data aggregation and merging. Uncertainty need not be off-putting as even incomplete uncertainty budgets add value to any observation. This paper aims to provide a starting point, or refresher, for researchers in the environmental sciences to make more complete use of uncertainty in their work.

## Article Highlights

- Presents worked examples of propagating uncertainty through the coarsening or merging of data
- Discusses how to avoid several common misconceptions in the analysis of environmental data
- Outlines best practices in the presentation of data to accurately represent uncertainty information

## Introduction

Uncertainty is an essential component of any scientific measurement, indicating the range of values that are consistent with a reported value, be it directly measured or derived from some calculation. Formally, BIPM et al. (2024, a collection of documents outlining best practice in metrology, the science of measurement) defines uncertainty as a “non-negative parameter characterising the dispersion of [values] being attributed to a measurand, based on the information used”, where the ‘measurand’ is the “quantity intended to be measured” in order to distinguish what one intended to measure from what was actually measured. They posit that uncertainty is distinct from ‘error’, which is formally the difference between a measurement and its reference or (typically unknowable) true value. BIPM notes that uncertainty is colloquially synonymous with ‘doubt’ and aims to describe the expected statistical behaviour of errors.

Space agencies now request that per-pixel estimates of uncertainty are provided for all ECVs they fund (e.g. ESA 2024), recognising that such information is necessary to understand complex systems with numerous natural and anthropogenic influences (Merchant et al. 2017). Information about the uncertainty in environmental data, even when incomplete, provides a deeper understanding of the data provided and focuses development of ECV algorithms (e.g. Rayner et al. 2014; Niro 2017; Popp and Mittaz 2022, Bulgin et al, 2025). Despite being included in any undergraduate course in the physical and life sciences, the treatment of uncertainty in environmental sciences literature can vary massively in complexity and rigour: from a simple calculation of the standard deviation of the difference between observations to fully characterised uncertainty trees rooted in fiducial reference measurements (e.g. Gal et al. 2024; Fernandes et al. 2014; Bulgin et al. 2016a; Mittaz et al. 2019).

This paper emerged from discussions at the International Space Science Institute’s workshop “Remote Sensing In Climatology— ECVs and their Uncertainties”. A recurring feature of the presentations was that ECV data users often lack the knowledge or confidence to utilise the uncertainty information available, mirroring the observations of ECV producers (such as aldred et al. 2023; Good et al. 2021). This manuscript aims to collect some practical advice on how uncertainty can be utilised when working with environmental data. These discussions are intended for researchers in the environmental sciences to complement the collection of examples of evaluating uncertainty, across all disciplines, that has been gathered in Part 5 of the Guide to Uncertainty in Measurement (BIPM et al. 2024).

Section 2 begins with a qualitative consideration of how uncertainty information can inform the interpretation of environmental data through the example of spatial variability around ocean fronts. Examples of common uncertainty calculations are provided in Section 3, including the aggregation of datasets. Factors that complicate data validation and assimilation are discussed in Section 4. Finally, Section 5 outlines several common misconceptions and strategies to avoid them, followed by concluding remarks.

## Considering Uncertainty

This section is aimed at users of environmental data. We qualitatively illustrate through an example why uncertainties matter and how to use uncertainty information to achieve a deeper understanding of data to make better decisions.

### Sampling Uncertainty when Examining Fronts

**Fig. 1:**
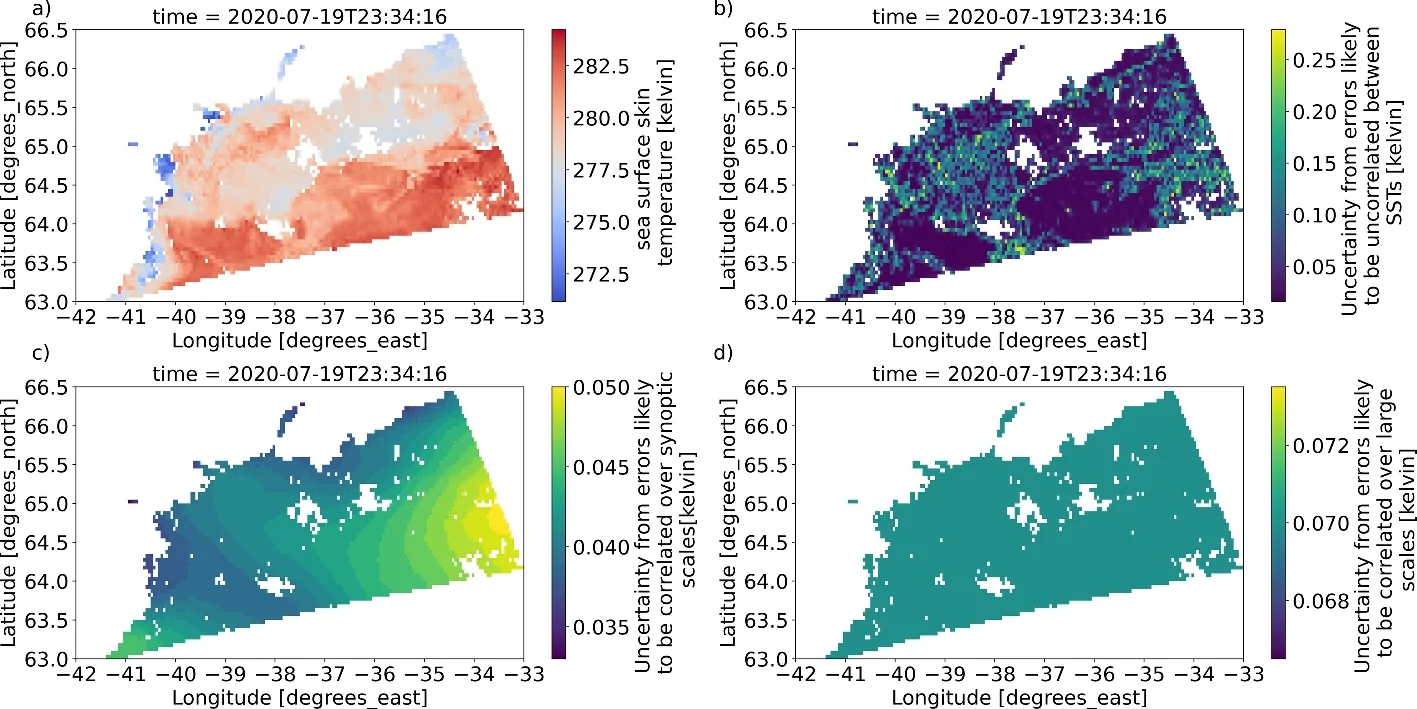
Retrieved sea surface temperature and associated uncertainties for the ESA CCI SST v3.0 L3U product 20200719233416-ESACCI-L3U_GHRSST-SSTskin-SLSTRA-CDR3.0-v02.0-fv01.0.nc. Subplots show: **a** SST, **b** uncertainties from error effects unlikely to be correlated between pixels, **c** uncertainties from error effects likely to be correlated between pixels on synoptic scales and **d** uncertainties from error effects likely to be correlated between pixels over large spatio-temporal scales. Retrievals are only made over the ocean. Missing pixels in the data field have been masked due to the presence of cloud

The European Space Agency (ESA) Climate Change Initiative (CCI) Sea Surface Temperature (SST) product provides not only a total uncertainty for each SST measurement but also a breakdown of this uncertainty value into its various components (Bulgin et al. 2016a; Embury et al. 2024). When analysed appropriately, the uncertainty budget gives the user a deeper insight into the dataset’s construction, enabling them to make informed choices on how to use and filter the data. Our example (Fig. 1) shows SST values along the south-east coast of Greenland, to the west of Iceland. The data are SSTs retrieved from the Sea and Land Surface Temperature Radiometer onboard the Sentinel-3A platform (SLSTR-A) on 19/07/2020 at 23:34. The data shown are Level 3 Uncollated (L3U), meaning that they are retrievals from a single satellite overpass, mapped onto a regular lat-lon grid (Embury et al. 2024).

The total uncertainty in the SST is constructed from three different uncertainty components and calculated by adding these in quadrature. These components are calculated independently in the product generation due to their different correlation length scales, ensuring correct propagation through the processing chain (Bulgin et al. 2016a), and are supplied as additional information to users. The first component is uncertainty that arises from error effects which are uncorrelated between pixels. For this product, these uncorrelated errors are primarily related to instrument noise (Bulgin et al. 2016a) and sampling uncertainty (Bulgin et al. 2016b). In the process of re-gridding the data from the instrument grid to a regular lat-lon grid, sampling uncertainty arises as the ocean surface is not fully sampled; some data are missing due to the presence of cloud obscuring the satellite’s view of the ocean.

The second uncertainty component is uncertainty that arises from errors correlated over synoptic scales (Bulgin et al. 2016a). The SST retrieval needs to account for the atmosphere (through which the ocean is viewed by the satellite), and this is represented using numerical weather prediction data that describe the surface temperature and total column water vapour. Errors in these prior values will lead to uncertainties correlated over synoptic scales (the scales on which the atmospheric conditions change). The third component is the large-scale, systematic uncertainty that arises from error effects correlated over large spatio-temporal scales (e.g. instrument specific calibration error effects applicable to whole satellite missions) (Bulgin et al. 2016a). In this example, these large-scale systematic uncertainties are dominated by instrument calibration errors.

The user can evaluate these uncertainty components alongside the SST data to better understand the drivers in the uncertainty variability. In this example, colder waters are found over the shelf seas surrounding Greenland (whose coast is the ragged edge on the left of the figures), with warmer water where the ocean deepens over the Irminger Sea (Chafik and Rossby 2019). This results in a number of SST fronts (strong gradients in SST) both along the shelf-edge and in regions of turbulent mixing between the shelf-sea and deeper waters (Fig. 1a). These SST frontal structures are most evident in the uncorrelated uncertainty component (Fig. 1b). This is because the magnitude of the sampling uncertainty is dependent on the underlying variability in the SST (Bulgin et al. 2016b). Where there are strong gradients in SST, calculating an area average in a gridded product containing some missing data gives a larger uncertainty than for regions where the SST is more homogeneous. The systematic uncertainty component (Fig 1c) is largest to the right of the image (just east of Iceland), with synoptic scale features evident in its variability. The large-scale systematic uncertainty component (Fig 1d) is consistent across the observed domain.

Understanding the uncertainty budget construction is of relevance to the user in deciding how to use and filter the data. Many users like to filter data on the basis of quality levels or thresholds and have a tendency to treat uncertainty estimates in the same way. However, large uncertainties are not indicative of bad data. If the user were to place a threshold on the total uncertainty to filter the data used in this example, they would exclude all areas of SST fronts from their analysis. In this example, where we plot an individual scene, this is perhaps obvious, but if users are interrogating a large dataset and placing a threshold on the total uncertainty, they may unwittingly introduce a bias in their results by preferentially screening out regions with greater SST variability.

Rather than a filter, an appropriate use of uncertainty is to weight inputs to a computation or analysis as uncertainty expresses the extent of doubt on a measurement. Basic methods to do so are outlined in Sect. 3. Data assimilation, a formal framework to weight data and its uncertainty against a model, is introduced in Sect. 4.3. Elsewhere in this issue, Formanek et al. (2025) discusses how to judge when uncertainty information may help or hinder various analyses and Gruber et al. (2025) introduces a framework for translating uncertainty into a form more useful for decision making.

### Ways of Representing Uncertainty

Uncertainty can be represented in either a parametric or in a nonparametric way. If the errors can be assumed to follow a probability distribution that is fully characterised by one (or more) parameter, that parameter can be used to represent the uncertainty. For example, ‘standard uncertainty’ is the most common representation of uncertainty when errors are assumed to be normally distributed, with zero mean, such as 16 ± 2 cm. The uncertainty, being the value after the ± sign, gives the standard deviation (σ) of that distribution. This implies that, if a measurement $x\, \pm \,\sigma$ were conducted repeatedly, 68% of observations would fall within the range $[x-\sigma , x+\sigma ]$.

If the probability distribution underlying the errors is more complex, non-symmetric, or unknown, a nonparametric representation of uncertainty may be needed, e.g. specifying a range of values that correspond to certain probabilities. Such ranges are commonly referred to as confidence intervals, such as $9.3\ (9.1-9.8)~\text {yr}$ (see Sect. 3.2 for details on their calculation). The range provided in the brackets indicates a range that is associated with a certain confidence level $\alpha \%$ that should be explicitly stated (typically 68, 90, or 95%). If the experiment was repeated many times, the true value would fall within $\alpha \%$ of the ranges so calculated (though any particular confidence interval either contains the true value or it does not).

It is crucial to understand that these two representations of uncertainty convey subtly, yet fundamentally, different information. Parametric uncertainty estimates are derived from calculations that involve prior knowledge about (at least partially) known error sources, whereas nonparametric interval estimates are derived from purely empirical sampling. In a very rough sense, confidence intervals are a frequentist representation of uncertainty and standard uncertainty is Bayesian (see Woolliams et al, in preparation, section‘DifferingviewsabouttheGUManditsapplication’).

It should be noted that this manuscript largely avoids the terms ‘random’ and ‘systematic’ as descriptions of error effects, for reasons discussed in Sect. 5.2. This diverges from the nomenclature of projects such as FIDUCEO (Mittaz et al. 2019) but is consistent in their intent. Further discussions concerning the communication of uncertainties and their different representations are provided in Sect. 5.3.

## Calculating Uncertainty

This section provides several quantitative examples of calculating the uncertainty on data or working with a provided uncertainty and discusses relevant practical issues and common misconceptions.

### Propagation of Uncertainty

The propagation of errors is widely taught in undergraduate courses, though it would be more accurately described as the propagation of *uncertainties*. This can be presented as ‘rules’ for sums, products, etc., but these derive from a general formula (Eq. 10 of JCGM 100:2008(E) BIPM et al. 2024) for the uncorrelated uncertainty of a variable *y* that is a function of a set of variables ${\textbf{x}} = (x_{1} ,x_{2} , \ldots ,x_{N} )$,

1 $$ \sigma ^{2} [y({\mathbf{x}})] = \sum\limits_{{i = 1}}^{N} {\left( {\frac{{\partial y}}{{\partial x_{i} }}} \right)^{2} } \sigma ^{2} (x_{i} ) $$

Here, σ(z) is the standard uncertainty on a variable *z* (which may itself be a function of other variables).

Application of this formula is straightforward, if not necessarily simple, for elementary functions. For example, the normalised difference vegetation index (NDVI) is a popular measure of the health and density of vegetation defined as,

2 $${\mathrm{NDVI}} = \frac{{\rho _{{{\mathrm{ir}}}} - \rho _{{{\mathrm{red}}}} }}{{\rho _{{{\mathrm{ir}}}} + \rho _{{{\mathrm{red}}}} }},$$

where ρ are reflectances measured by a satellite in an infrared ($\rho _\text {ir}$) or red ($\rho _\text {red}$) channel. If those measurements are assumed to be uncorrelated and suffer only measurement sources of error, the standard uncertainty on NDVI can be calculated as,

3 $$\begin{aligned} \sigma ^2(\text {NDVI})&= \left( \frac{ \partial \text {NDVI} }{ \partial \rho _\text {ir} } \right) ^2 \sigma ^2(\rho _\text {ir}) + \left( \frac{ \partial \text {NDVI} }{ \partial \rho _\text {red} } \right) ^2 \sigma ^2(\rho _\text {red}) \nonumber \\&= \left[ \frac{ 2\rho _\text {red} }{ \left( \rho _\text {ir} + \rho _\text {red} \right) ^2 } \right] ^2 \sigma ^2(\rho _\text {ir}) + \left[ \frac{ -2\rho _\text {ir} }{ \left( \rho _\text {ir} + \rho _\text {red} \right) ^2 } \right] ^2 \sigma ^2(\rho _\text {red}) \nonumber \\&= 4 \frac{ \rho _\text {red}^2 \sigma ^2(\rho _\text {ir}) + \rho _\text {ir}^2 \sigma ^2(\rho _\text {red}) }{ \left( \rho _\text {ir} + \rho _\text {red} \right) ^4 }. \end{aligned}$$

When the measurement equations cannot be expressed in terms of elementary functions (i.e. are difficult to write down comprehensibly), it is common to use finite differences to calculate the derivatives in (1),

4 $$ \sigma ^{2} [y({\mathbf{x}})] \approx \sum\limits_{{i = 1}}^{N} {\left[ {\frac{{y(x_{1} , \ldots ,x_{i}  + \delta , \ldots ,x_{n} ) - y(x_{1} , \ldots ,x_{n} )}}{\delta }} \right]^{2} } \sigma ^{2} (x_{i} ), $$

where δ is an arbitrary number that is small relative to the variables $x_i$ but large compared to the precision of the computer (and could vary with *i* if desired). More advanced approximations are available (e.g. Mickens 2015).

In practice, there will likely be some correlation between the radiances due to cross-talk, commonalities in the calibration method, stray light, or other imperfections in the measurement process (e.g. Mittaz et al. 2019; Holl et al. 2019; Ventress and Dudhia 2014). This tends to be overlooked when quantifying the correlation is difficult or impossible. Mittaz et al. (2019) explains that, when correlations are known, (1) can be generalised to,

5 $$  \sigma ^{2} [y({\mathbf{x}})] = \sum\limits_{{i = 1}}^{N} {\sum\limits_{{j = 1}}^{N} {\frac{{\partial y}}{{\partial x_{i} }}} } \frac{{\partial y}}{{\partial x_{j} }}\sigma ^{2} (x_{i} ,x_{j} ) $$

6 $$\equiv k^{T} Sk,$$

where $\sigma ^2(x_i,x_j)$ is the covariance of $x_i$ with $x_j$ and the second line expresses the sum as a product between the Jacobian $\textbf{k}$ (a column vector for which $k_i = \frac{ \partial y }{ \partial x_i}$) and the covariance matrix $\textbf{S}$ (for which $S_{ij} = \sigma ^2(x_i,x_j)$). The matrix formulation of this problem can be extremely useful when considering multiple error effects with different correlation length scales (Merchant et al. 2019). For the example of NDVI, (3) is amended to,

7 $$ \begin{aligned}   \sigma ^{2} (NDVI) =  & 4\frac{{\rho _{{red}}^{2} \sigma ^{2} (\rho _{{ir}} ) + \rho _{{ir}}^{2} \sigma ^{2} (\rho _{{red}} )}}{{\left( {\rho _{{ir}}  + \rho _{{red}} } \right)^{4} }} + \frac{{\partial NDVI}}{{\partial \rho _{{ir}} }}\frac{{\partial NDVI}}{{\partial \rho _{{red}} }}\sigma ^{2} (\rho _{{ir}} ,\rho _{{red}} ) \\     &  = 4\frac{{\rho _{{red}}^{2} \sigma ^{2} (\rho _{{ir}} ) + \rho _{{ir}}^{2} \sigma ^{2} (\rho _{{red}} ) - \rho _{{red}} \rho _{{ir}} \sigma ^{2} (\rho _{{ir}} ,\rho _{{red}} )}}{{\left( {\rho _{{ir}}  + \rho _{{red}} } \right)^{4} }}. \\  \end{aligned}  $$

Above, the correlation between the two error sources in the NDVI estimate reduces the estimated uncertainty, but this is not a general result. While it may be well-meaning to neglect the correlations in order to “avoid underestimation” in cases such as this, such uncertainties will be incorrect. Where it is impractical to write down the equations of a processing chain, or they are not susceptible to differentiation, Monte Carlo methods are available (see Supplement 1 of BIPM et al. 2024).

### Sampling Uncertainty and Confidence Intervals

Confidence intervals are an alternative means of representing the range of values consistent with a measurement. They are particularly useful where the distribution of an error is unknown or non-Gaussian. An example of this is estimation of trends or skill metrics, as these are subject to sampling uncertainties due to the difference between the spread of values in the sample and that of the population of all possible measurements. It is common practice to apply statistical tests to determine whether such a trend or skill estimates are ‘statistically significant’ (i.e. worthy of consideration or note). As will be discussed in Sect. 5.1, one should avoid such dichotomous and easily misinterpreted tests, if possible, and instead quantify the actual magnitude of the sampling uncertainties using confidence intervals.

Following Gruber et al. (2020), there are two methods for calculating a confidence interval. When the distribution of the measurand is not known, bootstrapping has been suggested as a nonparametric method for obtaining confidence intervals (Efron and Tibshirani 1986). Bootstrapping is a means of constructing empirical probability density functions (PDFs) by repeatedly resampling some original dataset, with replacement to preserve the sample size. In simple terms,

1. Resample the dataset of *n* data points by selecting *m* values from it at random, on each occasion selecting from all of the original data (a.k.a. with replacement so that a single value may appear repeatedly in the resample). Typically, n=m but *m* can be less than *n* when it is impractically large.
2. Use that resample to calculate the desired variable, such as a mean or a trend.
3. Store that value and repeat a large number of times to create an empirical PDF of the variable. A minimum of 1000 resamples is recommended (Efron and Tibshirani 1986).
4. Use that PDF to determine the confidence intervals.
  The most straightforward evaluation would be, for the 90% confidence level, to evaluate the 5th and 95th percentiles of that PDF (varying the values for other confidence levels as appropriate).
  That is only accurate to first order. Gilleland (2010) provides practical examples of the implementation of several other methods, aimed at the forecasting community.

If the variable of interest is believed to conform to some statistical distribution, *f*, only step 4 is necessary as the function can be used to evaluate the desired percentiles. For example, Gruber et al. (2020) defined the bias between two measures of soil moisture as $b_{xy} = \langle x \rangle - \langle y \rangle$, where $\langle \rangle$ denotes the mean of a dataset. The difference between two different estimates of the mean of a population is known to be distributed by Student’s *t* function, which has inverse cumulative distribution function $T_{n-1}^{-1}(a)$ giving the *a*th percentile of $b_{xy}$ that averages *n* measurements. Thus, the 90% confidence interval is,

8 $${\mathrm{CI}}_{{b_{{{\mathrm{xy}}}} }} = \left[ {b_{{{\mathrm{xy}}}} + \frac{ \in }{{\sqrt n }}T_{{n - 1}}^{{ - 1}} (0.05),b_{{xy}} + \frac{ \in }{{\sqrt n }}T_{{n - 1}}^{{ - 1}} (0.95)} \right],$$

where ϵ is an estimate of the uncertainty such as the root-mean square deviation (RMSD).

### Coarsening Datasets for Comparison with Other Data Sources

One of the most common manipulations of Earth observation datasets by data users is to apply some form of coarsening to relatively high-resolution data either to look at larger-scale averages (e.g. city-wide, regional or country-level values) or to enable comparison with other datasets (e.g. model outputs). The propagation of uncertainties through the coarsening step is often neglected, but should be applied to each uncertainty component in an uncertainty budget, with close attention paid to the correlation length scales of the various components. This section illustrates best practice for the procedure using land surface temperature (LST) data.

**Fig. 2:**
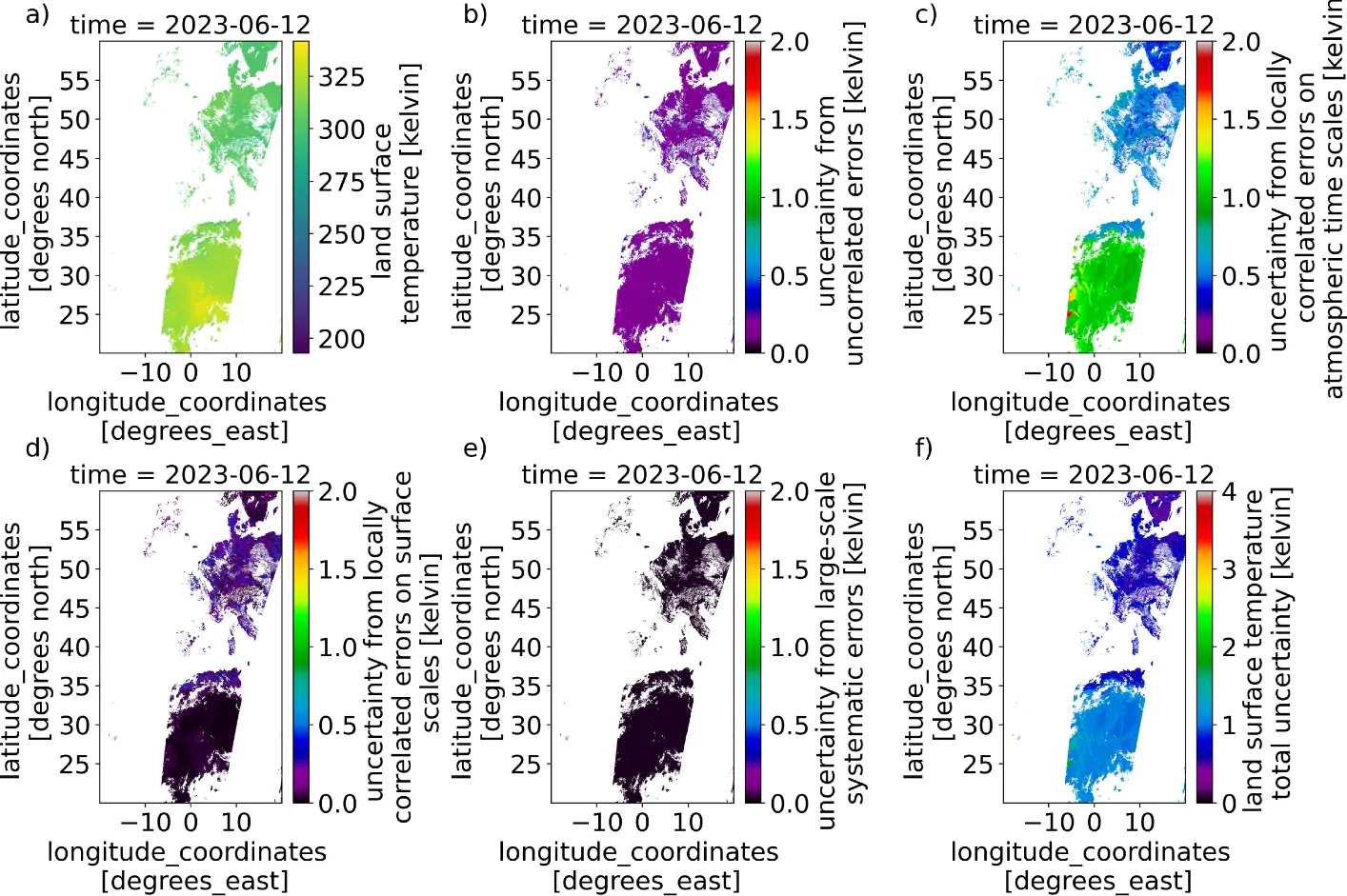
LST 0.01° daytime data product from Sentinel-3A SLSTR for 12/06/2023 showing **a** LST, **b** the uncorrelated uncertainty component, **c** the systematic uncertainty component correlated over atmospheric scales, **d** the systematic uncertainty component correlated over surface scales, **e** the large-scale systematic uncertainty component and **f** the total per-observation uncertainty. Data are taken from product file: ESACCI-LST-L3C-LST-SLSTRA-0.01deg_1DAILY_DAY-20230612000000-fv4.00.nc

LST products from the Sea and Land Surface Temperature Radiometer (SLSTR, described in Sect. 2.1) are provided at a resolution of 0.01°, an example of which is shown in Fig. 2 for a section of an orbit covering northern Africa and western Europe on 12/03/23. These datasets are provided with a full breakdown of uncertainty information into four different components: uncorrelated, locally correlated uncertainties on atmospheric scales, locally correlated uncertainties on surface scales, and large-scale correlated uncertainties. The per-pixel total uncertainty is also provided. Note that the uncertainty components (panels b-e) are all plotted on the same colour scale to highlight the difference in magnitude. The total uncertainty is the sum of the components (with the summation done in variance space).

**Fig. 3:**
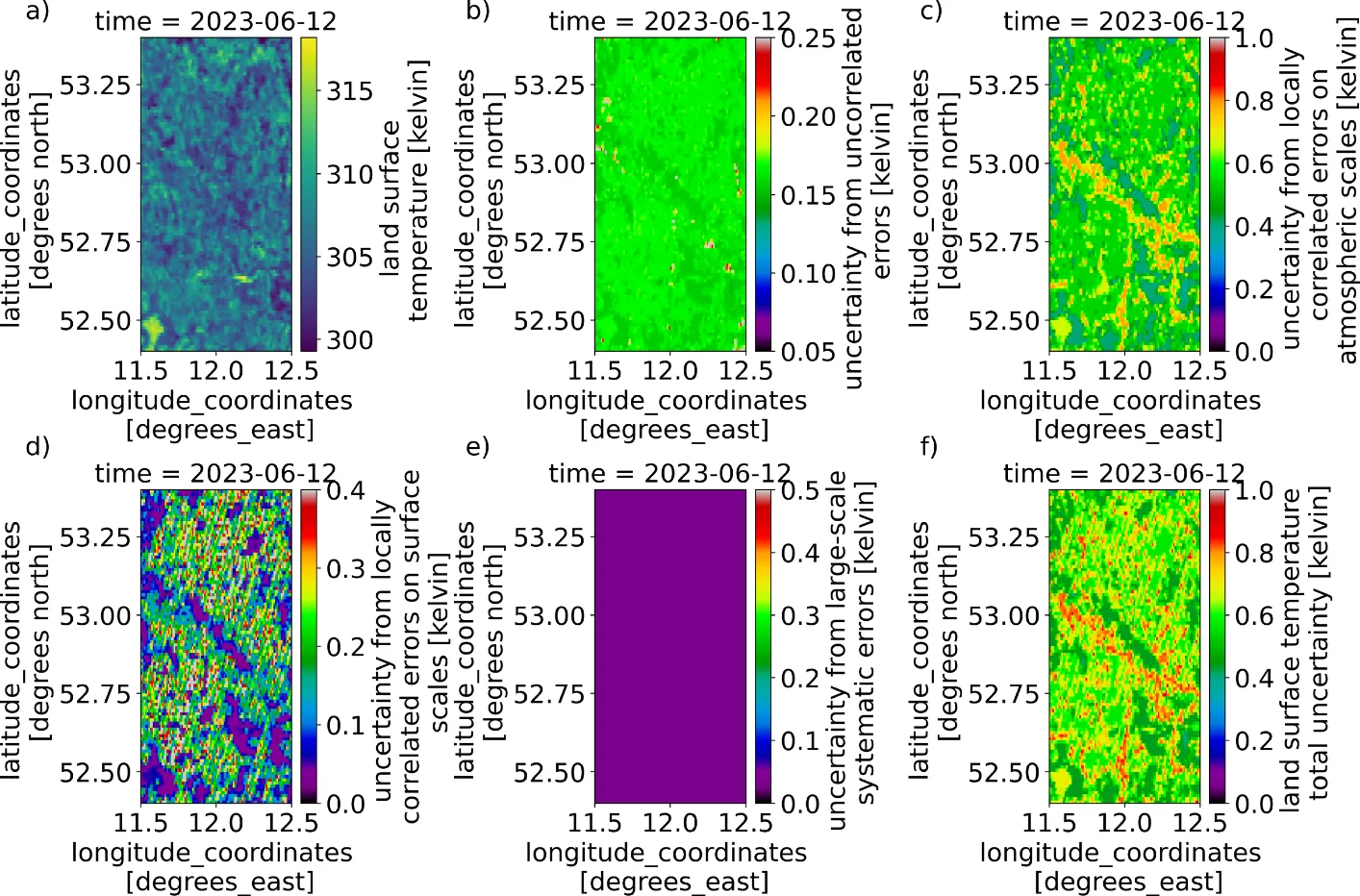
Subset of data from Fig. 2 located over northern Germany (52.4–53.4N, 11.5–12.5E). Panels show **a** LST, **b** the uncorrelated uncertainty component, **c** the systematic uncertainty component correlated over atmospheric scales, **d** the systematic uncertainty component correlated over surface scales, **e** the large-scale systematic uncertainty component and **f** the total per-observation uncertainty

Consider the case where we want to coarsen these data to a resolution of 0.05°. To illustrate this, we will focus on a small area in northern Germany of size 1°x1° (Fig. 3). Looking more closely, we can see differences in the spatial structure of the uncertainty components, reflecting their sensitivity to factors that govern their correlation length scales. For example, the atmospheric component is more smoothly varying than the surface component as the latter is correlated with land cover (biome).

To propagate the uncertainties, we require equation 5:, which can be expanded as:

9 $$  \sigma ^{2} [y({\mathbf{x}})] = \sum\limits_{{i = 1}}^{n} {\left( {\frac{{\partial y}}{{\partial x_{i} }}} \right)^{2} } \sigma ^{2} (x_{i} ) + 2\sum\limits_{{i = 1}}^{{n - 1}} {\sum\limits_{{j = i + 1}}^{n} {\frac{{\partial y}}{{\partial x_{i} }}} } \frac{{\partial y}}{{\partial x_{j} }}\sigma ^{2} (x_{i} ,x_{j} ).  $$

The first term relates to the propagation of the uncertainties that are uncorrelated (see also Eq. 1), the second term relates to the correlated uncertainties (see also Eq. 6), and *n* is the number of measurements combined in this grid cell.

To coarsen the data to 0.05°, we apply an arithmetic average to the retrieved LST under the assumption that the LST is variable over the coarser domain and all observations carry equal weight:

10 $$\langle {\mathrm{LST}}\rangle = \frac{1}{n}\sum\limits_{{i = 1}}^{n} {\mathrm{L}} {\mathrm{ST}}_{i} .$$

Differentiating this equation with respect to LST shows that the sensitivity is $\frac{1}{n}$. Inserting this into Eq. 9 yields:

11 $$  \sigma ^{2} [{\mathrm{LST}}({\mathbf{x}})] = \sum\limits_{{i = 1}}^{n} {\left( {\frac{1}{n}} \right)^{2} } \sigma ^{2} (x_{i} ) + 2\sum\limits_{{i = 1}}^{{n - 1}} {\sum\limits_{{j = i + 1}}^{n} {\frac{1}{n}} } \frac{1}{n}\sigma ^{2} (x_{i} ,x_{j} ).  $$

For the uncorrelated uncertainty component, $\sigma ^2(x_i,x_{j \ne i}) = 0$ and this equation simplifies to:

12 $$ \sigma _{{{\mathrm{uncor}}}} [{\mathrm{LST}}({\mathbf{x}})] = \frac{1}{{\sqrt n }}\sqrt {\frac{{\sum\limits_{{i = 1}}^{n} {\sigma ^{2} } (x_{i} )}}{n}} . $$

The $\frac{1}{\sqrt{n}}$ scaling is applied under the assumption that the input uncertainty is a constant value and the uncertainty is uncorrelated between pixels. The uncertainties are channel specific, specified in brightness temperature space and then propagated through the LST retrieval equation. Dependencies of coefficients within this retrieval equation on land cover, fractional vegetation and total column water vapour mean that the resultant $\sigma _{uncor}^2(x_i)$ values are not constant, and *n* is added to the denominator of 12 on the right hand side to take the average. For the fully correlated case, $\sigma ^2(x_i,x_{j \ne i}) = \sigma (x_i)\sigma (x_j)$ and Eq. 11 simplifies to:

13 $$ \sigma _{{{\mathrm{cor}}}} [{\mathrm{LST}}({\mathbf{x}})] = \sqrt {\frac{{\sum\limits_{{i = 1}}^{n} {\sigma ^{2} } (x_{i} )}}{n}} . $$

This applies to both the large-scale correlated component and the component that is locally correlated on atmospheric scales. This is because the given spatial correlation length scale for the atmospheric component is 5 km (Steinke et al. 2015; Vogelmann et al. 2015), which is equal to the resolution of the new grid (and so the uncertainty is fully correlated over this domain).

**Fig. 4:**
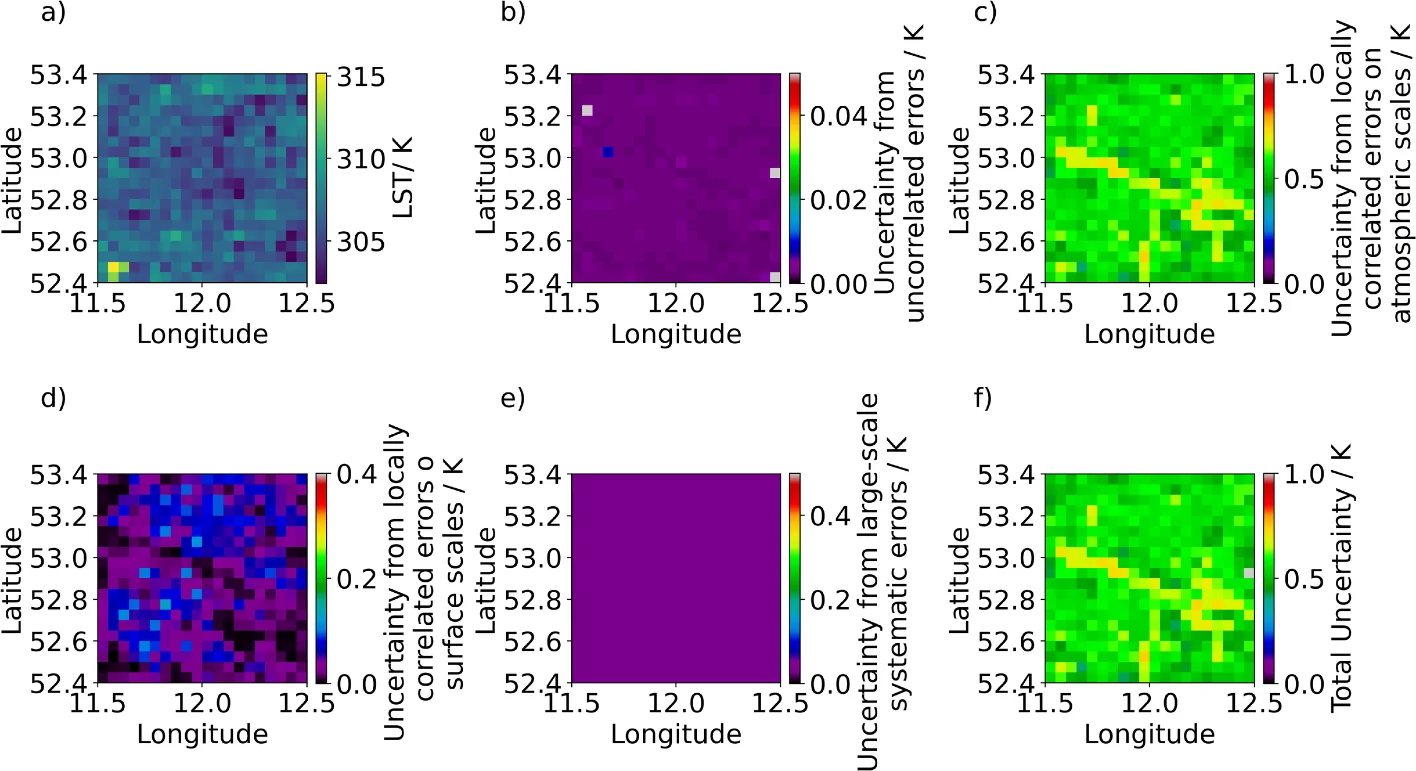
Data from Fig. 3 coarsened to a resolution of 0.05°. Panels show **a** LST, **b** the uncorrelated uncertainty component, **c** the systematic uncertainty component correlated over atmospheric scales, **d** the systematic uncertainty component correlated over surface scales, **e** the large-scale systematic uncertainty component and **f** the total per-observation uncertainty

Propagation of the surface component is more complex as the correlation differs between observation pairs. The information provided with the product states that the uncertainties are correlated for pixels of the same biome, with a spatial correlation length scale of 5 km (Ghent et al 2017). The propagation is done using the matrix form of the equation, explicitly constructing the covariance matrix for each 0.05° grid cell according to the biome distribution. The propagated uncertainties for each component are shown in Figure 4.

The total uncertainty is the sum of the components, in quadrature:

14 $$ \sigma [{\mathrm{LST}}({\mathbf{x}})] = \sqrt {\sigma _{{{\mathrm{unc}}}}^{2} ({\mathbf{x}}) + \sigma _{{{\mathrm{atm}}}}^{2} ({\mathbf{x}}) + \sigma _{{{\mathrm{surf}}}}^{2} ({\mathbf{x}}) + \sigma _{{{\mathrm{sys}}}}^{2} ({\mathbf{x}})} . $$

Coarsening of any dataset should be approached in this way, taking into account the correlation length scale of each component of the uncertainty budget. Note that this example uses complete data, e.g. there are no missing observations due to cloud. If sampling was incomplete, an additional sampling uncertainty term should also be calculated.

### Merging Datasets with Robust Uncertainty

When multiple measurements of the same quantity are available, it may be desirable to merge them into a single estimate. For example, Gruber et al. (2017) merge 11 soil moisture products into a single time series using the standard uncertainty as a weighting factor to average *m* observations of the same measurand *x* by,

15 $$\begin{aligned} x_\text {agg}&= \frac{ \sum _{i=1}^m x_i / \sigma ^2(x_i) }{ \sum _{i=1}^m 1 / \sigma ^2(x_i) } \end{aligned}$$

16 $$\begin{aligned} \sigma ^2(x_\text {agg})&= \left( \sum _{i=1}^m \frac{ 1 }{ \sigma ^2(x_i) } \right) ^{-1}. \end{aligned}$$

This is an application of weighted least squares, currently used in the generation of the ESA CCI soil moisture climate data records (Gruber et al. 2019). Triple collocation analysis (e.g. Virtanen et al. 2018; Gruber et al. 2016) is applied to obtain robust and mutually consistent estimates of the standard uncertainties (and hence weights) of different satellite soil moisture products (an outline of the method can be found at https://pytesmo.readthedocs.io/en/latest/examples/triple_collocation.html; last accessed 4 March 2025). Note that this method neglects any correlation within and between errors and assumes that all measurements are samples from the same distribution (neglecting error effects such as sampling). These would add terms to the uncertainty budget (c.f. Eq. 13 vs. Eq. 12). Despite the absence of robust estimates of potential error covariances, this weighted least squares implementation was shown to yield merged soil moisture time series that perform better than the individual input products, and those obtained from a more simplistic ordinary least squares implementation (i.e. an unweighted average), highlighting how even incomplete uncertainty budgets can add value to data and its analysis.

### Merging Datasets with Inconsistent Uncertainty Budgets

The methods of previous sections rely on the uncertainty estimates of the input variables being fit-for-purpose (i.e. accurate and precise), although it is still possible for there to be aspects of an uncertainty budget that it is difficult to fully quantify (Mittaz et al. 2019). The following discussion is adapted from Popp et al. (2024), which outlines the creation of a merged dataset of aerosol optical depth (AOD) for the Copernicus Climate Change Service. AOD is the integral of extinction and absorption due to aerosols through a vertical column of the atmosphere. This adaptation is intended as a demonstration of the practical considerations involved in the use of uncertainty and is not an endorsement of this specific method for dataset merging. The fitness of a dataset’s uncertainty can be judged by comparison against reference observations, and a detailed review of methods to achieve such evaluation is provided elsewhere in this issue by Verhoelst et al. (2025).

AERONET (Aerosol Robotic Network, Holben et al. 1998) is a network of sun-photometers that automatically locate the sun and measure AOD from the attenuation of direct illumination. This is assumed to have an uncertainty of $\sigma (\tau _a)=0.01$, which is about an order of magnitude smaller than the uncertainties typically reported by satellite AOD products. Once AERONET observations are co-located with the satellite data, there will be *N* pairs of observations $\tau _s(i)$ and $\tau _a(i)$. From those, the ‘expected discrepancy’ $u_x(i)$ and the ‘bias-corrected difference’ $d_x(i)$ are calculated as,

17 $$\begin{aligned} u_x(i)&= \sqrt{\sigma ^2[\tau _s(i)] +\sigma ^2[\tau _a(i)]} \end{aligned}$$

18 $$\begin{aligned} d_x(i)&= \tau _s(i) - \tau _a(i) - \frac{1}{N} \sum _{j=1}^N \tau _s(j) - \tau _a(j). \end{aligned}$$

A correction factor is then applied to the satellite uncertainties to ensure that their distribution (i.e. the spread of expected discrepancies) matches the distribution of observed errors (i.e. the spread of bias-corrected differences),

19 $$\begin{aligned} \sigma _*^2(\tau _s) = \left( \frac{ \langle |d_x| \rangle }{ \langle |u_x| \rangle } \right) ^2 \sigma ^2(\tau _s). \end{aligned}$$

For a practical example, consider a synthesised set of six observations by three unbiased instruments:

0. An AERONET sun-photometer with error of 0.01;
1. A satellite imager with error 0.06 that is accurately represented by the uncertainty; and
2. A second imager with similar error but incorrect uncertainty estimate of 0.03.

From this information, $\sigma ^2(\tau _a) = 10^{-4}$, $\sigma ^2(\tau _1) = 3.6\times 10^{-3}$, and $\sigma ^2(\tau _2) = 9\times 10^{-4}$. Hence, $u_1 = 0.061$ and $u_2 = 0.032$. Table 1 then presents simulated data from the sun-photometer (column $\tau _a$), the well-characterised imager (column $\tau _1$), the poorly characterised imager (column $\tau _2$), and the application of Eq. 15 to those data. For example, in the second row,

20 $$\begin{aligned} \tau _\text {agg}&= \frac{ \tau _1 / \sigma _1^2 + \tau _2 / \sigma _2^2 }{ 1 / \sigma _1^2 + 1 / \sigma _2^2 } \nonumber \\&= \frac{ 0.002 / 3.6\times 10^{-3} + 0.031 / 9\times 10^{-4} }{ 1 / 3.6\times 10^{-3} + 1 / 9\times 10^{-4} } \nonumber \\&= 0.0252. \end{aligned}$$

By construction, each instrument provides an accurate estimate of the mean of the complete set of six observations. Hence, columns $d_{1/2}$ are simply the difference between columns $\tau _{1/2}$ and $\tau _a$. Then, the correction factor for satellite 1 is $\langle | d_1 | \rangle / u_1 = 0.060 / 0.061 = 0.948$ and for satellite 2 it is $\langle | d_2 | \rangle / u_2 = 0.060 / 0.032 = 1.91$. (The corrected uncertainties are coincidentally both $\sigma _*(\tau _{1/2}) = 0.057$.)

**Table 1:** Demonstration of the calculation of a weighted mean for six simulated observations by a sun photometer ($\tau _a$) and two satellite products ($\tau _{1,2}$). If the uncertainty on the second satellite is underestimated, $\tau _\text {agg}$ gives the weighted mean of the observations. After correcting the uncertainties, a new aggregation $\tau _\text {agg}'$ provides a more precise estimate as measured by the RMSD (bottom row)

	True value	$\tau _a$	$\tau _1$	$\tau _2$	$\tau _\text {agg}$	$|d_1|$	$|d_2|$	$\tau _\text {agg}'$
	0.04	0.024	0.088	0.098	0.096	0.064	0.074	0.093
	0.06	0.067	0.002	0.031	0.025	0.065	0.036	0.016
	0.08	0.096	0.108	0.017	0.035	0.012	0.079	0.063
	0.10	0.097	0.167	0.053	0.076	0.070	0.044	0.110
	0.16	0.151	0.043	0.129	0.112	0.108	0.022	0.086
	0.22	0.225	0.252	0.332	0.316	0.027	0.107	0.292
Mean	0.11	0.110	0.110	0.110	0.110	0.058	0.060	0.110
RMSD		0.011	0.065	0.063	0.055			0.051

We quantify the precision of the two merged datasets using a RMSD against the known ‘true’ value in the bottom row. The aggregated dataset $\tau _\text {agg}$ somewhat improves upon each individual dataset (RMSD of 0.055 compared to 0.065 or 0.063), while the corrected aggregation $\tau _\text {agg}'$ is an improvement upon all three (RMSD 0.051). The process to correct these uncertainties, being based on noisy data, is only approximate—the ‘accurate’ uncertainties are improperly reduced by 5% while the ‘inaccurate’ uncertainties are only increased by 90% when they should be doubled. A sample of thousands of data points would be preferable to provide greater resilience against outliers.

To repeat, this is only one way of utilising imperfect uncertainties. Popp et al. (2024) goes on to consider correction factors that vary with $u_x$, expecting the errors to increase for larger signals. Further, the corrections could have been applied to variances rather than standard deviations. A more metrological approach would use this validation of the uncertainties to revise the estimates themselves, but empirical correction factors of this form can be useful when confronted with practical realities.

## Using Uncertainty

This section discusses the role of uncertainty in the comparison of different measurements of the same measurand.

### Comparing Single Datum

**Fig. 5:**
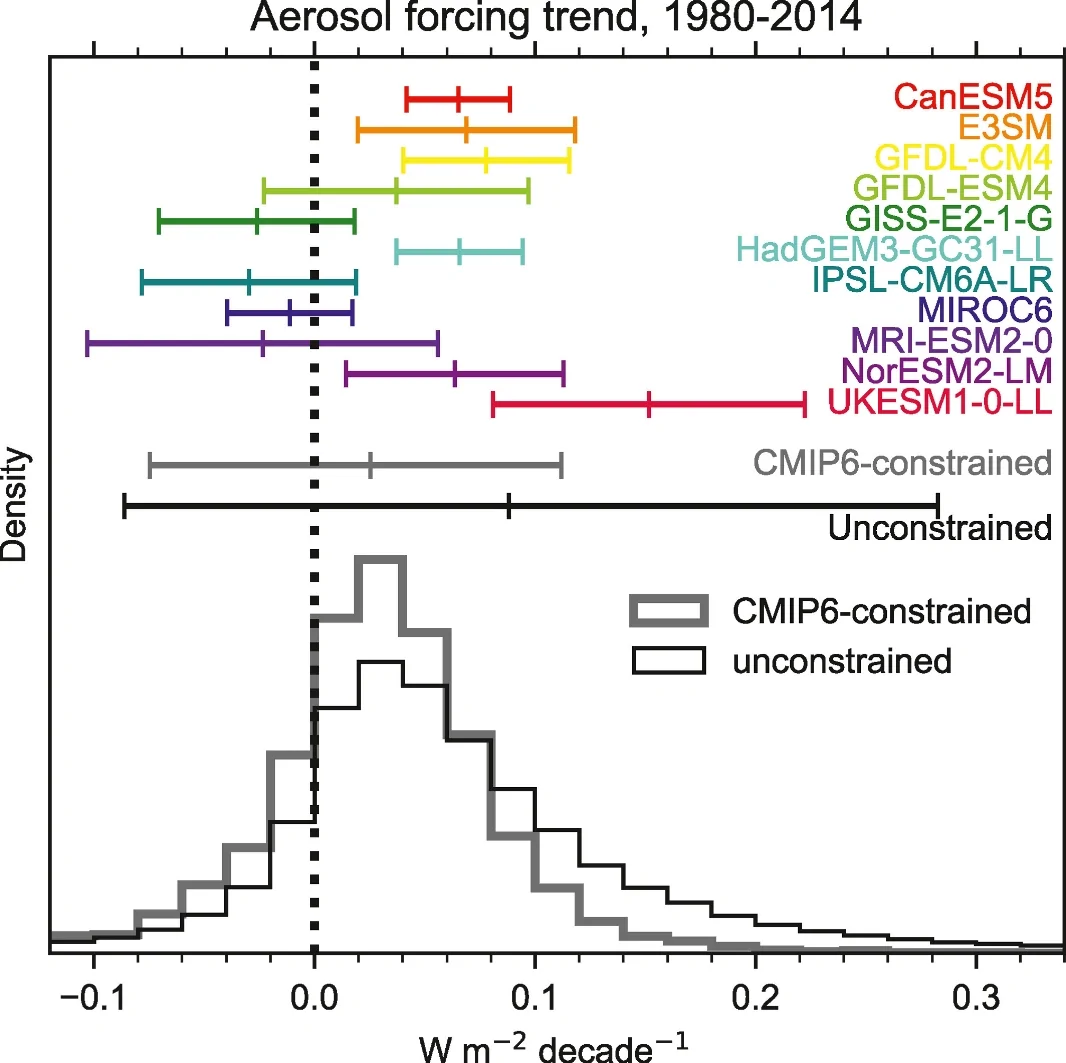
Trends of linear aerosol forcing for 1980–2014 in various CMIP6 models (coloured whiskers), with 90% confidence intervals (reproducing Fig. 7 of Smith et al. (2021)). The histograms summarise the output of Monte Carlo simulations of the same trend with two different weightings

The most elementary use of uncertainty information is to determine if two measurements of the same quantity are consistent with each other. Physical science textbooks that use the term ‘consistent’ appear to avoid defining the term (e.g. Hughes and Hase 2010), but usage appears similar to “likely to be of the same value”. Statisticians reserve the term for describing estimators that converge to a single value as the sample size increases (Dodge 2003). In the former sense, a pair of observations $x_1\pm \sigma _1$ and $x_2\pm \sigma _2$ are said to be consistent if their difference is smaller than the combination of their uncertainties, namely if $|x_1 - x_2| < \sqrt{\sigma _1^2 + \sigma _2^2}$. A similar comparison can be made using confidence intervals (i.e. two results are consistent if their confidence intervals overlap). While possibly having qualitative value, comparisons of this form both misrepresent the uncertainty information available and are liable to misunderstanding.

Comparisons of confidence intervals are common for metrics that summarise a complex system, particularly in the comparison of climate models. An example is reproduced from Smith et al. (2021) in Fig. 5, which compares trends in aerosol radiative forcing between different models and a distribution of that variable estimated with a statistical model. The authors complement the set of intervals by aggregating them into a PDF at the bottom of the figure, which combines the individual uncertainty estimates into useful and easily understood information.

By graphically representing uncertainties with error bars, the most intuitive interpretation is that the ‘true’ value of the measurand is within the ranges of both measurements. The danger is that where the ranges barely overlap (corresponding to small certainty in their combination), the apparent range for the true value is small (corresponding to high certainty). Further, for confidence intervals, overlap can be achieved simply by increasing the confidence level, which disguises the decreased certainty in the comparison. As such, this qualitative comparison is easier to understand if the name is inverted—testing *inconsistency*. If a pair of observations differ by more than the combination of their uncertainties, they are inconsistent and additional information will be needed to assess the measurand.

To comment on the agreement of observations requires formally testing the hypothesis that two measurements are drawn from the same sample. If an analysis merely comments on the consistency of observations, rather than merging the data into a single estimate (see Sect. 3.4), it may imply that the uncertainties provided are considered to be incomplete or incompatible in some way, such that only a qualitative statement is possible.

### Comparing Data

When multiple data points are available, the above comparisons can be generalised. Called ‘validation’, this is an essential step in the creation of an ECV record, demonstrating the utility of the data by comparison against independent observations. The essential components of a validation are to:

1. Co-locate the datasets;
2. Collect sufficient data to achieve statistically robust results; and
3. Create a scatter plot and summary statistics.

Each of these steps involves a number of practical and statistical considerations, with numerous common misapprehensions. Loew et al. (2017) is recommended for a comparison of methodologies across different ECVs. The validation process can also be applied to uncertainty estimates themselves (e.g. Bulgin et al. 2016a; Sayer et al. 2020), and the methods of that process (and how they differ to that outlined below) are reviewed elsewhere in this issue by Verhoelst et al. (2025).

#### Co-locate Datasets

Co-location is a procedure to harmonise datasets, identifying sets of observations of the same circumstances (Merchant et al. 2017). The most straightforward case would be two identical sensors stationed side-by-side that record with the same periodicity. In that simple experiment, co-location would simply involve applying quality control to each dataset (i.e. removing spurious or contaminated results) and pairing the observations that are closest in time. Things are rarely that simple in practice.

Consider two identical radiometers observing the same patch of ground in order to determine its temperature. The instruments will view slightly different scenes as they are in different positions —a tree may shade more of the patch seen from one vantage causing that instrument to consistently report a lower temperature. This will result in differences between the observations in addition to those that the experiment is attempting to estimate, introducing additional uncertainty into the validation (e.g. Ermida et al. 2014). Anisotropy of the surface can produce similar effects when seemingly minor changes in viewing geometry result in substantial differences in (say) emissivity of the patch observed by each instrument. It will not always be possible to minimise these uncertainties, such as when the instruments have different spectral responses. Like the shade of the tree before, if some aspect of the scene emits light in a part of the spectrum only detected by one instrument, there will be a difference between the observations. This may be consistent over time (such as a rock) or not (such as a transient puddle). Hence, it is important to carefully consider precisely what is measured by each instrument when designing co-location. It may be necessary to apply corrections to the data to avoid conducting an ‘apples-to-oranges’ comparison of different measurands.

That example is still simpler than most validations conducted on ECVs. More typically, a satellite swath is compared to a surface station, aircraft track, or other satellite. This requires matching data in both time and space, which results in multiple possible pairings. Is it better to compare observations that occurred simultaneously but with a large spatial separation, coincident measurements separated in time, or to average all of the available data? That question would ideally be resolved by considering the spatio-temporal covariance expected in the quantity being measured to determine comparison scales over which there is a reasonable expectation that the two observations are representative of each other. For example, a grassland may be fairly homogeneous when viewed at a resolution of hundreds of metres but exhibit sharp discontinuities at metre-scales that can resolve shrubs. Equivalent issues may occur in the time domain. For example, nitrogen dioxide concentrations exhibit a diurnal cycle, which might imply that observations should be closely matched, but if the cycle can be measured then a correction may provide sufficient accuracy to permit validation (e.g. Compernolle et al. 2020).

When data on the spatio-temporal covariance are unavailable or incomplete, models can provide valuable insight. Schutgens et al. (2017) used high-resolution simulations of aerosol loading to determine that the difference between two simulated observations is minimised when they are within 4–6 hr, depending on the size of the model grid. The precise values will depend on the variable being assessed and exactly what information is sought. In practice, co-location tends to select thresholds (such as 30 min and 25 km), average all observations within those limits, and then compare those aggregated data points between instruments. This approach is largely practical —a straightforward way to reduce the volume of data to be handled —but will reduce the impact of stochastic error sources (which may be an advantage or a disadvantage, depending on the intent of the validation). As covariances are rarely characterised at sufficient scale, thresholds are determined by trial-and-error, such that re-using the parameters of a previously published validation is acceptable for a small or preliminary study.

#### Collect Sufficient Data

A major concern of validation is completeness and representativeness —are the data provided by co-location an unbiased sample of all possible circumstances? The essential requirements are that (i) sufficient observations are considered such that an additional one is unlikely to substantively change the results, and (ii) the observations are a representative sample of the circumstances one intends to observe. These concepts do not have consistent terminology in the environmental sciences.

If one searches the internet for “minimum sample size”, there are numerous posts and calculators that recommend between 30 and 100 values. This inflexible approach has been repeatedly critiqued (e.g. Sertdar et al. 2020; Wutich et al. 2024) and, though far from the only discussion of this misconception, Chakrapani (2011) outlines how such calculations assume independent, normally distributed sampling with replacement. For realistic data (in his case survey responses, but the argument applies to any somewhat correlated data, including most ECV data products), a larger sample is necessary to achieve normality. Thus, during validation there is no specific number of observations that achieves robust results. The goal is collect sufficient data that it is believed unlikely that the results of the analysis would change if more data were added (i.e. the statistical definition of ‘consistency’, Dodge 2003).

It is necessary to consider the distribution of ‘errors’ realised during the experiment, i.e. to plot a histogram of “measurement – reference” for the co-located data to inspect their distribution. In our opinion, it is not necessary to achieve strict normality (for which many tests are available in Yap and Sim 2011) but merely to have a distribution that is qualitatively symmetric and peaked around a central value such that the standard deviation is a useful representation of the majority of the data (even if not necessarily representative of the tails of the error distribution). Where this is not the case, the validation should identify auxiliary variables with which to subdivide the co-located data in order to achieve symmetric distributions within each division. These variables are expected to represent processes that introduce error into either dataset (or their comparison), such as the zenith angles of the sun and sensor, ambient humidity, cloud fraction, or wind speed.

Validation should include observations across as much of the domain as possible for the measurand and any parameters important to its derivation. This involves considering sampling across:

- the full range of the measurand, capturing the expected minima, maxima, and mode;
- different times of day, seasons, or the solar cycle (as appropriate);
- the oceans and continents of the Earth;
- surface types such as deep or shallow ocean, snow- or ice-covered land, prairie, forest, desert, urban areas, and so on; and
- observation conditions or confounders such as viewing zenith angle, sea-surface roughness, or loading of stratospheric aerosols.

It will be infeasible to sample across all of these in any one dataset. Regardless, a validation that neglects an important dimension (such as the common example of only evaluating over a single city, region, or country) may omit a significant portion of the uncertainty budget. The important consideration is the domain over which the validation is needed—a local validation does not provide confidence in a global product. The impacts of sampling and representativeness errors are complex (Bulgin et al. 2022), but empirical estimates can often provide a useful first guess before a more detailed assessment is performed, such as preparing an uncertainty tree (Mittaz et al. 2019). As validation datasets are increasingly used to train machine learning algorithms, it is vital that co-location datasets capture a more complete summary of the measurand’s domain as users may not be aware of the limited domain of preliminary validation. Otherwise, the outputs are liable to ‘out-of-training data’ errors, whereby an empirical model behaves unpredictably when presented with circumstances beyond those that were used in its creation.

The inability to observe all relevant conditions introduces a representativeness error into both the validation and the datasets themselves. A widespread example is ‘clear-sky bias’. Remote sounding of the Earth’s surface can typically not be done in the presence of cloud. ECVs where cloud is expected to impact the value, such as surface temperature in the infrared, where direct sunlight provides a substantial input of energy (Ermida et al. 2019), will therefore be biased to only describe clear-sky conditions relative to the all-sky population that would be seen using data at microwave wavelengths or by an in situ reference sensor.

Another limitation is the availability of reference observations. Reference sites are much more common in the northern hemisphere for most ECVs, with regions such as the Southern Ocean systematically undersampled, making it difficult to characterise processes concentrated in the global south. The poles are typically difficult to work in during winter, such that reference observations of (say) sea ice thickness are concentrated in the spring despite being known to be unrepresentative of other times of year (e.g. Rostosky et al. 2018). These and more examples (e.g. Dorigo et al. 2021), as well as methods to alleviate the uncertainties, are discussed in section 3 of Langsdale et al. (2025).

**Fig. 6:**
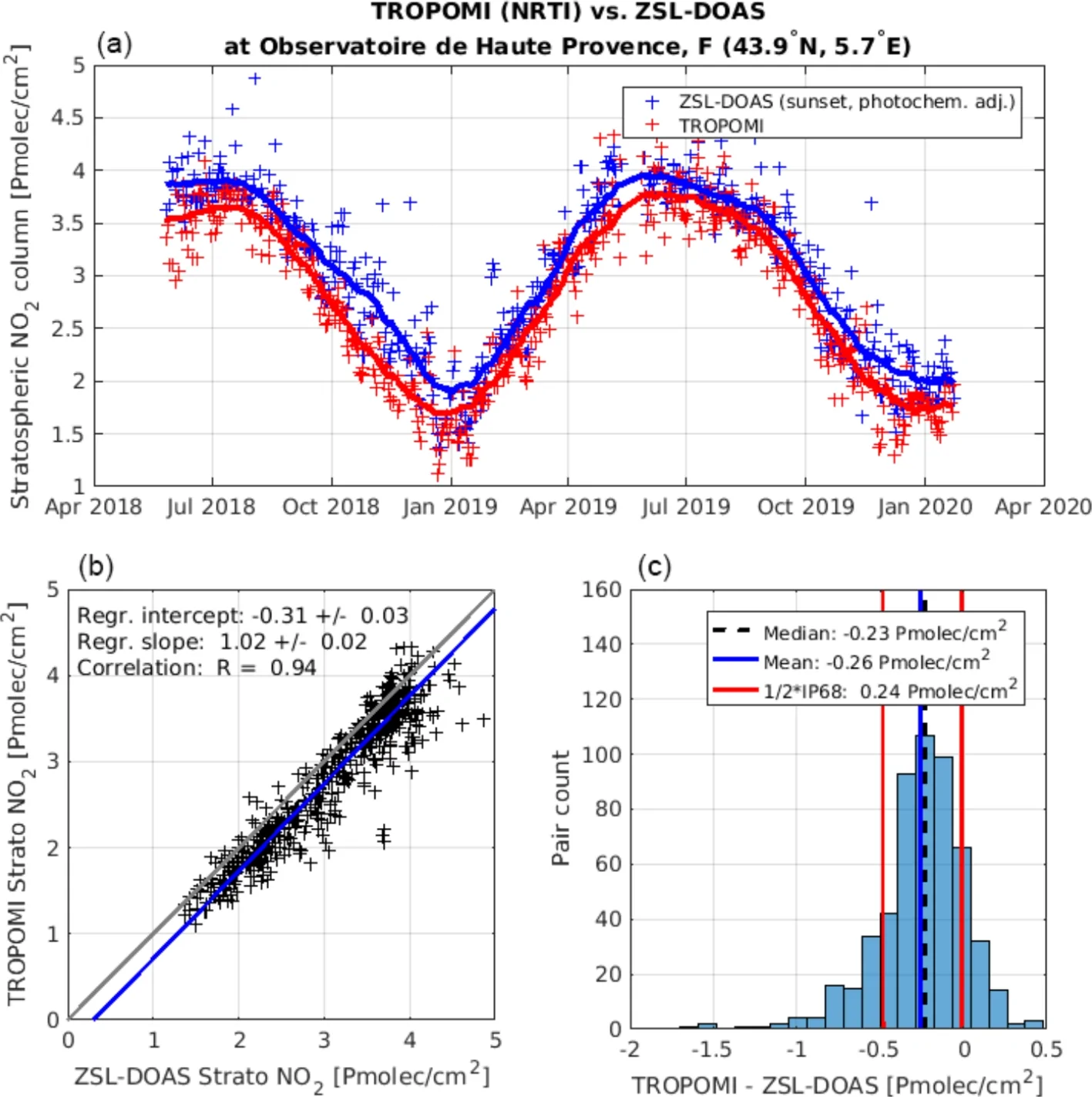
**a** Time series of stratospheric NO_2_ column data from the TROPOMI instrument (red) co-located with ground-based measurements zenith-scattered light differential optical absorption spectroscopy (ZSL-DOAS, blue). Solid lines represent 2-month running medians. **b** Scatter plot and **c** histogram of the differences between them, superimposed with several statistical measures of the agreement between data. Reproduces Fig. 4 of Verhoelst et al. (2021)

#### Statistical Summary

Once data have been co-located and its representativeness assured, they are statistically assessed to determine the nature of agreement between the datasets (Loew et al. 2017). Though many types of analysis are available (such as triple collocation, see Stoffelen 1998), the most common presentation of validation results in the environmental sciences is a linear regression. An exemplary form of this is shown in Fig. 6, reproducing the validation of stratospheric NO_2_ column density from Verhoelst et al. (2021). Panel (a) shows a time series of the two datasets, helping the reader to easily identify temporal properties such as instrument drift or seasonal influences. While time series are useful for qualitatively illustrating a product’s performance (as they can be produced without co-location), they are more of a verification than a validation, in the terminology of Loew et al. (2017) as they do not provide a quantitative assessment of a dataset’s utility. In addition, this plot could be improved by scaling the lengths of the crosses to show the uncertainty in the data, though this would produce a cluttered figure for large datasets. Panel (c) provides the histogram requested in the previous section, presenting a sufficiently symmetrical distribution of discrepancies despite a long negative tail. The choice of bin size is usually ad hoc, but objective methods are available to select the number of evenly spaced bins (e.g. Freedman and Diaconis 1981). (An informal discussion of several options can be found at https://numpy.org/doc/stable/reference/generated/numpy.histogram_bin_edges.html; last accessed 4 March 2025.)

Panel (b) provides the traditional scatter plot, with the ‘reference’ observation plotted along the *x*-axis. Such figures provide a simple illustration of the dataset for the avoidance of, for example, Simpson’s Paradox (whereby two correlated datasets will appear uncorrelated if combined; Simpson 1951). There are several simple choices that can improve the accessibility and utility of such a plot. As before, it could scale the crosses to express the uncertainty on each dataset, but this can rapidly make it impossible to distinguish data. Once the number of observations exceeds a hundred or so, it is advisable to switch from plotting each datum separately to either a hex-plot, two-dimensional histogram, or other diagram that uses shading to convey the distribution of data among the variables. Otherwise, it is possible to mask the presence of a significant bias where data overlap (e.g. Fig. 12 of Hirschi et al. 2023). (The rainbow colour map should not be used in such plots to reduce the risk of over-interpretation of the data and to increase accessibility to colour-blind (or other) readers, see Crameri et al. 2020)

The figure also summarises the linear correlation between the datasets, with the slope and intercept of a linear regression on panel (b). As both datasets have uncertainty, this should be performed using orthogonal distance regression rather than elementary linear regression (e.g. scipy.odr rather than numpy.polyfit or scipy.stats.linregress). Quantitative metrics of the goodness of fit should also be included, such as the linear correlation coefficient *R* and RMSD (though error is sometimes used in place of deviation and this is suboptimal; see Sect. 5.2). The variables plotted should be chosen to spread the data as evenly as possible across the domain. For example, a log-normally distributed variable should be plotted on a log-scale to conform with the assumptions of the regression method (Sayer and Knobelspiesse 2019). The importance of both visualising the co-location data and providing summary statistics is humorously illustrated by ‘The Datasaurus Dozen’, a set of twelve datasets with identical regression statistics but wildly different distributions (Matejka and Fitzmaurice 2017).

### Data Assimilation

The term ‘data assimilation’ describes methods whose aim is to optimally integrate imperfect model simulations with observations that are subject to errors (Lahoz and Schneider 2014). One can think of it as using real-world observations to “pull model simulations in the right direction”, but also as using model simulations to interpolate between discontinuous observations. Either way, all data assimilation methods are based on weighted averaging, aiming to create a merged estimate with uncertainties lower than any single input (Gelb 1974). To achieve this, weights need to be derived from the model and observational uncertainties following least-squares theory.

Data assimilation techniques differ in what assumptions they make about error (correlation) structures, and how these structures are taken into consideration. The most relevant distinction is that between ‘variational data assimilation’ (Le Dimet and Talagrand 1986) and ‘sequential data assimilation’ (Bertino et al. 2003), which will be explained in the following subsections, followed by a discussion of common difficulties when using uncertainty information contained in ECV data products for data assimilation purposes.

#### Variational Data Assimilation

Variational data assimilation assumes no spatial and/or temporal dependency between neighbouring model estimates and thus can update multiple model estimates simultaneously by minimising a cost function *J* of the form:

21 $$J({\mathbf{x}}) = \frac{1}{2}(\mathbf{x} - \mathbf{x}_{b} )^{T} {\mathbf{P}}^{{ - 1}} (\mathbf{x} - \mathbf{x}_{b} ) + \frac{1}{2}(\mathbf{y} - {\mathbf{H}}\mathbf{x})^{T} {\mathbf{R}}^{{ - 1}} (\mathbf{y} - {\mathbf{H}}\mathbf{x}),{\text{ }}$$

where $\textbf{x}_b$ is the model state (‘background’) vector; $\textbf{y}$ is the vector of the observations that are mapped into the model space using the observation operator $\textbf{H}$; and $\textbf{P}$ and $\textbf{R}$ are the model and observation error covariance matrices, respectively. The diagonals of those matrices summarise the uncertainties in $\textbf{x}_b$ and $\textbf{y}$, while their off-diagonals give estimates of error covariance.

Variational data assimilation is widely used in numerical weather prediction, especially for re-analysis problems to obtain the best possible state of the atmosphere (Bannister 2017). It is common to distinguish between three-dimensional (3D-Var; Courtier et al. 1998) and four-dimensional (4D-Var; Lorenc 2003) variational data assimilation problems. The former simultaneously updates horizontal and vertical model fields at a single updating time step, whereas the latter considers observations taken over an extended period of time to create a complete spatial and temporal reanalysis of the model forecasts.

#### Sequential Data Assimilation

Sequential data assimilation accounts for cases where the uncertainty of a model simulation at time *t* depends on the forecast uncertainty of the previous time step t-1. In such cases, the ‘optimal’ weight for assimilating an observation at time step *t* changes after another observation at time step t-1 has been assimilated. Consequently, to maintain optimal uncertainty reduction, state updating has to be done sequentially, accounting for the change in model uncertainty at each time step.

The most common method to do this is the Kalman Filter (Evensen 2003), which calculates optimal merging weights for state updating (the ‘Kalman gain’) whenever an observation is available and then applies the law for the propagation of uncertainty (see Sect. 3.1) to calculate the impact of the state updates on model background uncertainty. This is typically written as:

22 $${\mathbf{K}}_{t} = {\mathbf{P}}_{t}^{ - } ({\mathbf{P}}_{t}^{ - } + {\mathbf{H}}_{t} {\mathbf{R}}_{t} {\mathbf{H}}_{t}^{{\mathrm{T}}} )^{{ - 1}} .$$

Here, $\textbf{P}_t^-$ is the model background uncertainty and $\textbf{K}_t$ is the Kalman gain, which is equivalent to the observational weight derived according to generalised least squares theory, used to update the model background state vector $\textbf{x}_t^-$ as:

23 $$\begin{aligned} \textbf{x}_t^+ = \textbf{x}_t^- + \textbf{K}_t(\textbf{y}_t - \textbf{H}_t\textbf{x}_t), \end{aligned}$$

where $\textbf{x}_t^+$ is the updated model state vector. The updated model uncertainty $\textbf{P}_t^+$ follows by applying Eq. (6) to Eq. (23) as:

24 $$\begin{aligned} \textbf{P}_t^+ = (\textbf{I}-\textbf{K}\textbf{H}_t)\textbf{P}_t^-. \end{aligned}$$

Finally, $\textbf{P}_t^+$ has to be evolved through the model to calculate the model background uncertainty at the next updating time step:

25 $$\begin{aligned} \textbf{P}_{t+1}^- = f(\textbf{P}_{t}^+). \end{aligned}$$

The function *f* depends on the functional form of the used model, which is often difficult to calculate given the complex, nonlinear nature of many common Earth system models. Therefore, a modification to the Kalman filter is often used, most commonly the Extended Kalman Filter (De Rosnay et al. 2013), which uses local approximations to the modelling functions, and the Ensemble Kalman Filter (Evensen 2003), which uses Monte Carlo simulations to evolve model uncertainty.

#### Practical Issues when Using Uncertainty in Data Assimilation

Whatever the method, data assimilation always applies some sort of weighted averaging where the weights should be inversely proportional to the uncertainties of the estimates that are being integrated (i.e. the model simulations and the observations). When assimilating ECVs, one should thus be able—in theory—to use the uncertainty estimates that are provided with the ECV data as input to the assimilation system (provided, of course, that the uncertainty estimates are a realistic representation of the ECV errors).

This is not commonly done. Most published approaches ignore the uncertainty estimates provided and do one of the following: (i) manually tune the data assimilation parameters until they achieve satisfactory improvements (e.g. by evaluating the data assimilation performance against reference data, Heyvaert et al. 2023; ii) estimate model and observation estimates themselves from reference data (Crow and van den Berg 2010); or (iii) optimise the assimilation system using internal diagnostics, i.e. variables of the system that should follow an expected behaviour if the uncertainties were parametrized correctly, such as the time series of the differences between model forecasts and observations (Desroziers et al. 2005). Correlations between observations are removed by thinning the dataset (Hoffman 2018).

Note that the disregard of ECV uncertainty estimates in these approaches is not a result of ignorance but of an unavoidable issue: ECV uncertainty estimates characterise the errors of the observations with respect to a measurand that is different from that of the model into which the observations should be assimilated. That is, ECV uncertainties describe the deviations from the true state of the observed variable within the satellite footprint and a wavelength-dependent signal penetration depth, while the assimilation requires the deviations from the true state averaged across the modelling grid cell with an arbitrarily chosen modelling layer depth. It is common to resample observations to the modelling grid before assimilation, but the observational uncertainties are not usually updated to account for this as it would require estimates of representativeness uncertainties, which are usually not available and can be difficult to obtain.

A second important issue is that data assimilation weights are derived from the *relative* magnitude of the uncertainties in the observation to the model simulations. That is, even if ECV uncertainties were known exactly, the uncertainties in the model simulations need to be known as well in order to calculate optimal weights. Unfortunately, estimating uncertainties for models simulations is considered even more difficult than for observations (Kumar et al. 2022). This is because most models used in Earth system science are highly complex, and predicting uncertainties in their simulations would require not only reliable estimates of the uncertainties in all model forcing variables and model parameters, but also estimates of error correlations across space, time, and model variables, and estimates of representation uncertainty, i.e. the uncertainty associated with the inaccurate physical representation of the real world as well as the uncertainty related to the spatial and temporal scale mismatch between the model forcing and the model output grid.

As a consequence, instead of attempting to obtain rigorous estimates of model and observation uncertainties that account for error correlations and representativeness errors with respect to the modelling grid, it is usually more fruitful to derive empirical approximations of model uncertainties and then calibrate simplistic observation uncertainties to achieve satisfactory performance in the data assimilation system when evaluated against real-world reference data.

This does not mean that one should give up on detailed uncertainty modelling. In fact, it suggests that it is even more important to properly understand all relevant sources and interactions of errors in order to determine simple yet robust uncertainty representations that account for the most relevant uncertainty components, correlation length scales, and representativeness.

## Communicating Uncertainty

While most researchers have been exposed to uncertainty throughout their education, a number of misconceptions and misunderstandings are widespread. This section discusses several of these of relevance to providers of data in the environmental sciences and makes recommendations on how to avoid them.

### Hatching, Trends, and Significance

The concepts of Sect. 4.1 can also be applied to the comparison of a dataset with itself, for example, to assess the presence of trends within a time series. The calculation of trends and their uncertainties is covered in detail by Gobron et al. (2025) in this issue and so will not be discussed here. However, significance and the associated *p*-values are a widely misinterpreted and misused concept, to the extent that the American Statistics Association (ASA) was moved to state six principles of best practice in the use of *p* values (Wasserstein and Lazar 2016). The first of these, “*p* values can indicate how incompatible the data are with a specified statistical model”, is pertinent to a widespread use of significance in the environmental sciences and the use of hatching on plots.

**Fig. 7:**
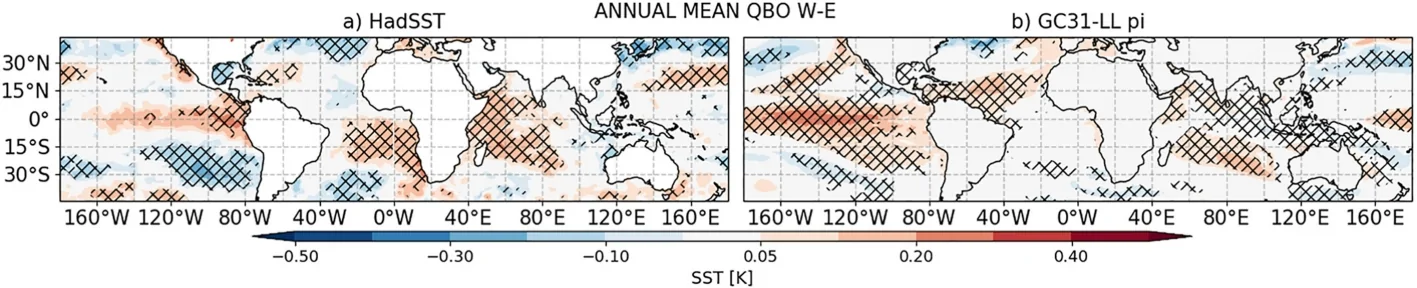
Differences between the west and east modes of the quasi-biennial oscillation (QBO) in annual mean sea-surface temperature (in K) from **a** the HadSST dataset and **b** a pre-industrial control simulation of the Unified Model GC31-LL pi. Hatching denotes significance at the 95% confidence level. Reproduces Fig. 1 of García-Franco et al. (2023)

When presenting trends on a map, it is common practice to add hatching to direct the reader’s attention to ‘significant’ trends (i.e those for which the *p*-value is below some threshold). For example, the quasi-biennial oscillation (QBO) is a mode of variability in the stratosphere. Fig. 7 reproduces a plot from García-Franco et al. (2023) showing the difference in sea-surface temperature between the extremes of that teleconnection. In panel (a), the hatching emphasises the negative values over the southern Pacific while de-emphasising the positive values over the tropical Pacific.

As indicated by the second ASA principle (“*p* values do not measure the probability that...the data were produced by random chance alone”), areas without hatching are not noise to be ignored. In this example, the *p* value gives the plausibility of the statistical model “data that shares a single mean”. As Greenland et al. (2016) discusses, (i) this calculation relies on several assumptions, such as independence of observations, that tend to be violated by ECV data; (ii) the calculation tests a single, stated hypothesis (i.e. no change in a time series) as opposed to rejecting a set of plausible hypotheses (e.g. linear change, quadratic change, step change) as is the goal of exploratory data analysis; and (iii) it is possible to directly evaluate the hypothesis of interest (e.g. the measurand changes linearly with time) and doing so would be more useful as it requires the investigator to clearly state what they aim to find. They state, “Any opinion offered about the probability, likelihood, certainty, or similar property for a hypothesis cannot be derived from statistical methods alone. In particular, significance tests and confidence intervals do not by themselves provide a logically sound basis for concluding an effect is present or absent with certainty or a given probability."

The intention of hatching is to direct attention to regions for which the reported trend is inconsistent with the uncertainty reported on the data (or with natural variability where that is considered to be more substantial, such as Fig. 14 of Swaminathan et al. 2022). In other words, the hatching should indicate the detection limit for trends in those observations. This distinction is subtle—that ‘insignificant’ trends are not necessarily non-existent but merely excessively uncertain. Conversely, ‘significant’ trends are not necessarily real, particularly when drawn from data with an incomplete uncertainty budget (see also Gobron et al, 2025), or may be negligibly small but merely known to be so with great confidence. García-Franco et al. (2023) avoided this mistake in their discussion of Fig. 7, which described how the observational record (panel a) resembles an El Niño response due to aliasing between two teleconnections in recent years. (An El Niño response is a dipole over the western Pacific, half of which is not hatched in the diagram).

Regardless, it would be more useful to base hatching on, for example, trends that are larger than two standard uncertainties of the underlying data or for which the confidence interval does not include zero. Uncertainty directly relates to the intention of the figure, examines the limitations of the data rather than a statistical model, and sidesteps the extensive argument about significance tests.

### Error and Uncertainty

When communicating uncertainty, it is important to avoid using the term ‘uncertainty’ in a manner synonymous with ‘error’ because they are two distinct concepts. Recall that the definitions of the terms in Sect. 1: an error is the deviation of an actual measurement from the unknown true state of the measurand, whereas the uncertainty describes the distribution of all possible errors associated with the measurement. This matters because one can estimate uncertainties but not errors. Language thus needs to be chosen accordingly: while averaging measurements really does reduce errors, we can only predict how much it reduces the uncertainty. For example, it is meaningful to evaluate uncertainty components, but not error components. And, probably most commonly misused, one can propagate uncertainties, but not errors.

Special attention also needs to be given to the distinction between ‘random’ and ‘systematic’ errors, which is often made in elementary statistics. As elegantly discussed in the ‘Handling error correlation’ section of Woolliams et al (in preparation), these are rather limiting descriptions of real behaviours. Random errors are usually considered entirely independent (e.g. thermal noise), whereas systematic errors are considered to follow a common, predictable pattern (e.g. the result of an incorrect calibration constant). In reality, the degree of dependence between errors will often be a combination of both due to, e.g. correlated errors. This determines, for example, by how much the uncertainty can be reduced upon averaging (see Sect. 3). Moreover, sources of uncertainty that are ‘random’ at one point (say stochastic noise on a radiance measurement) can become ‘systematic’ at another (such as when that measurement is used to calibrate a sequence of observations by another sensor). Hence, while ‘random error’ or ‘systematic error’ are phrases likely to be recognised by the reader, it is more informative to emphasise the *source* of the errors (e.g. stochastic versus systematic effects), and the expected degrees and dimensions of error correlation.

Finally, a point of semantics. It is correct to speak of correlated errors but not of correlated uncertainties because the term ‘uncertainty’ refers to a probability distribution (in one manner or another) and the value of uncertainty is some parameter of that distribution (e.g. ‘standard uncertainty’ refers to a normal distribution and its value is the standard deviation of that distribution). The correlation exists between the realisation of an error effect through a course of measurements, not between the parameters used to describe those errors. Unfortunately, the distinction between realised errors and descriptive uncertainties has not been extended to the terminology for covariance such that ‘correlated uncertainty’ is typically used as a shorthand for ‘the component of uncertainty that arises from correlated error effects’ despite the inconsistency of applying an adjective which describes an error effect to an uncertainty.

### Notation

**Table 2:** Methane lifetime due to chemical losses, soil uptake and total atmospheric lifetime based on CMIP6 multi-model analysis, and bottom-up and top-down methane budget estimates, reproducing Table 6.2 of Szopa et al. (2021). Values in parenthesis show the minimum and maximum range while uncertainties indicate a ±1 standard deviation

Study	Total chemical lifetime (years)	Soil lifetime (years)	Total atmospheric lifetime (years)
Stevenson et al. (2020)	8.3 (8.1–8.6)	160	8.0 (7.7–8.2)
Bottom-up	8.3 (6.2–9.8)	166 (102–453)	8.0 (6.3–10.0)
Top-down	9.7 (9.4–10.5)	135 (116–185)	9.1 (8.7–10.0)
AR6 assessed value	9.7±1.1	135±44	9.1±0.9

Recall from Sect. 2.2 that uncertainty may be represented in a parametric (e.g. standard uncertainty) or nonparametric (as confidence intervals) way. Recent reports of the Intergovernmental Panel on Climate Change (IPCC 2023) make extensive use of confidence intervals due to the asymmetric distribution of the errors associated with many metrics of climate change. That report often uses a 95 % confidence interval, which would be similar to a width of 2σ for the Gaussian standard uncertainty (e.g. $16\ (12-20)~\text {cm}$ for the value given at the start of this section) *only if the source of error is normally distributed*. Hence, it is important to check the conventions being used when comparing printed results to avoid apples-to-oranges comparisons.

An illustration of the potential difficulty in comprehending data comparisons is Table 6.2 of Szopa et al. (2021), which compares various methane lifetimes (reproduced here as Table 2). An inattentive reader may assume the table provides confidence intervals, but the caption states that the ranges are actually minimum and maximum (and so not conveying formal uncertainty information). A hint to this comes from the mixture of intervals with standard uncertainties and the lack of a per cent sign within the caption. This emphasises both the importance of clearly stating what uncertainty information is provided (as done in this example) and reading that statement when first encountering data.

The interface between uncertainty and the colloquial meaning of ‘confidence’—the certainty one has in a course of action—is discussed in Gruber et al. (2025). The need for a more consistent use of statistical and metrological terminology within the Earth sciences is presented in Strobl et al. (2024).

## Conclusions

Uncertainty provides the context necessary to understand and utilise data. Without uncertainty, observations cannot be appropriately compared, combined, or propagated into subsequent calculations. Even incomplete uncertainty budgets can provide value to both users and producers of data. Large uncertainties, either relatively or absolutely, are not an indication of suspect or unusable data, and this paper has outlined how removing data from an analysis based on the magnitude of uncertainty can errantly remove regions of interest.

The uncertainty in most ECVs is itself uncertain and being open, honest, and comprehensive in the measurement, analysis, and quality assurance procedures applied to a dataset is essential in ensuring that uncertainty can be communicated, utilised and improved. Evaluations of uncertainty should consider empirical methods to assess unquantified terms. A commonly overlooked term is measurand differences (colloquially called ‘apples-to-oranges’ comparisons), being the distinctions between ostensibly equal variables caused by differences in resolution, timing, spectral range, etc. They can be approximated through modelling. These methods are of particular importance to data assimilation, where input uncertainties should be defined with respect to the model’s definition of measurand rather than the measurand definition in the assimilated observations.

The assessment of the covariance structure of ECVs is too often overlooked. It supports the selection of collocation criteria during validation, trend analysis, change detection, assimilation, aggregation, and more. Covariance can be determined from modelling studies, climatology captured by long-term observations, and targeted in situ sampling.

Several errant statistical shorthands and rules-of-thumb remain in use and this paper joins the many calls to improve statistical literacy and practice. Uncertainty is a measure of doubt, such that two observations with non-overlapping error bars can be said to be inconsistent with each other but evaluating their consistency requires additional information or analysis. Significance does not comment on the probability that a result was produced by chance and, more generally, does not signify scientific relevance. We reflect that the creators of such tests considered them but one of many tools from which a statistical argument should be crafted.

Best practices in the presentation of uncertainty information were discussed to ensure accurate and effective communication. Foremost, data producers should liaise with each other and their user communities to identify the expected lexicon for uncertainty. Where practical concerns limit the ability to follow their advice, short user guides should explain the presentation in plain language. Validation studies should illustrate that data are drawn from a single population by accounting for confounding variables. Where that is impractical and errors are unknown, confidence intervals provide a sensible first estimate of uncertainty. When researchers wish to focus attention on some subset of a noisy data field, hatching should be based on uncertainty or a clearly stated hypothesis test rather than the significance level. As environmental data are rarely compared to a laboratory-standard reference, analyses of linear regression should account for uncertainty in all inputs, specifically avoiding simple linear regression. Validation should strive to include as many real-world conditions as practical to demonstrate that data are fit for any purpose to which a reasonable user might apply it.

This work’s central thesis is that uncertainty need not be off-putting. Uncertain data are not bad. To extend the examples presented here, readers are encouraged to consult Part 5 of BIPM et al. (2024). While comprehensive uncertainty budgeting may appear to require immense resources or delicate knowledge across all areas of science, a practical approach of honesty and best effort can achieve most of what uncertainty is attempting to communicate while laying the foundations for future improvement. Remember that to err is human; to measure, uncertain.
